# Laparoscopic-assisted Ex Vivo Reconstruction of Renal Artery Aneurysm with Internal Iliac Artery and Auto-transplantation

**DOI:** 10.7759/cureus.3611

**Published:** 2018-11-19

**Authors:** Abdul Ahad Rana, Brendan H Dias, Santosh Olakkengil, Christine Russell, Shantanu Bhattacharjya

**Affiliations:** 1 Surgery, Royal Adelaide Hospital, Adelaide, AUS; 2 Urology, Royal Adelaide Hospital, Adelaide, AUS

**Keywords:** renal artery aneurysm, ex vivo reconstruction, laparoscopic assisted ex vivo aneurysmectomy

## Abstract

Renal artery aneurysms (RAA) represent a complex and an often incidentally found disease commonly treated with endovascular approaches. In cases where in situ approaches are unsuitable, laparoscopic-assisted ex vivo repairs offer significant advantages during and post-surgery. We present a case of a female patient who presented with a long-standing right-sided flank pain. She was diagnosed with bilateral asymptomatic RAAs positioned well into the hilum, therefore making in situ repair infeasible. A laparoscopic-assisted ex vivo repair of the renal artery was performed using a graft from the internal iliac artery, which is a novel approach.

## Introduction

Renal artery aneurysms (RAA) are infrequently encountered [1], with a prevalence of 0.15% to 0.1% in the general population [2]. Majority of the RAAs are discovered incidentally when the patient undergoes diagnostic imaging studies for other unrelated complaints such as hypertension or back pain. While the issue of treatment of RAAs has been surrounded by significant debate, there is agreement that pregnant patients, patients with aneurysms with a diameter greater than 2 cm, or with thrombus must definitely be treated [2]. While previously open surgical repair and endovascular approaches have been commonly used, one of the surgical treatment options that is gaining increasing popularity is the laparoscopic-assisted ex vivo aneurysmectomy [3]. This is because of its low morbidity and mortality as well as acceptable rates of consequent renal function and blood pressure control [4]. The ex vivo repair allows significant benefits especially for RAAs whose branches are not anatomically suitable for endovascular treatment or those that occur in the hilar or distal renal artery. In this report, an ex vivo reconstruction and auto-transplantation of a bilateral renal artery aneurysm has been presented, and the diagnosis, treatment, and clinical outcomes of this approach have been discussed.

## Case presentation

**Figure 1:**
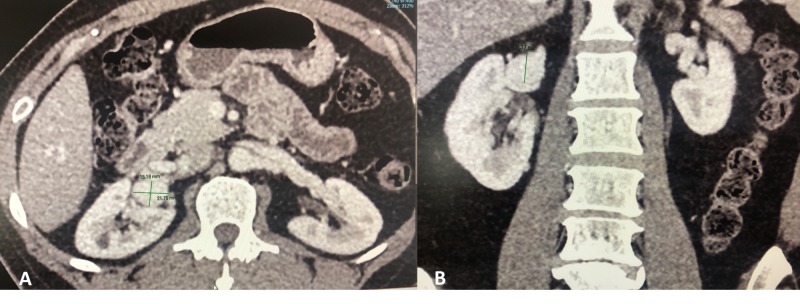
Axial (A) and coronal (B) CT images showing a right renal artery aneurysm measuring 21 x 15 x 17 mm CT: computed tomography

We present a case of a 53-year-old female who presented with a long-standing history of right flank pain. Computed tomography (CT) scan showed bilateral renal artery aneurysms measuring less than 1 cm on the left, and 2.1 x 1.5 cm and 1.7 cm on the right (Figure 1). As is a typical case, these RAAs were asymptomatic and because of their position in the hilum, these were anatomically suitable for an in situ repair. 

**Figure 2:**
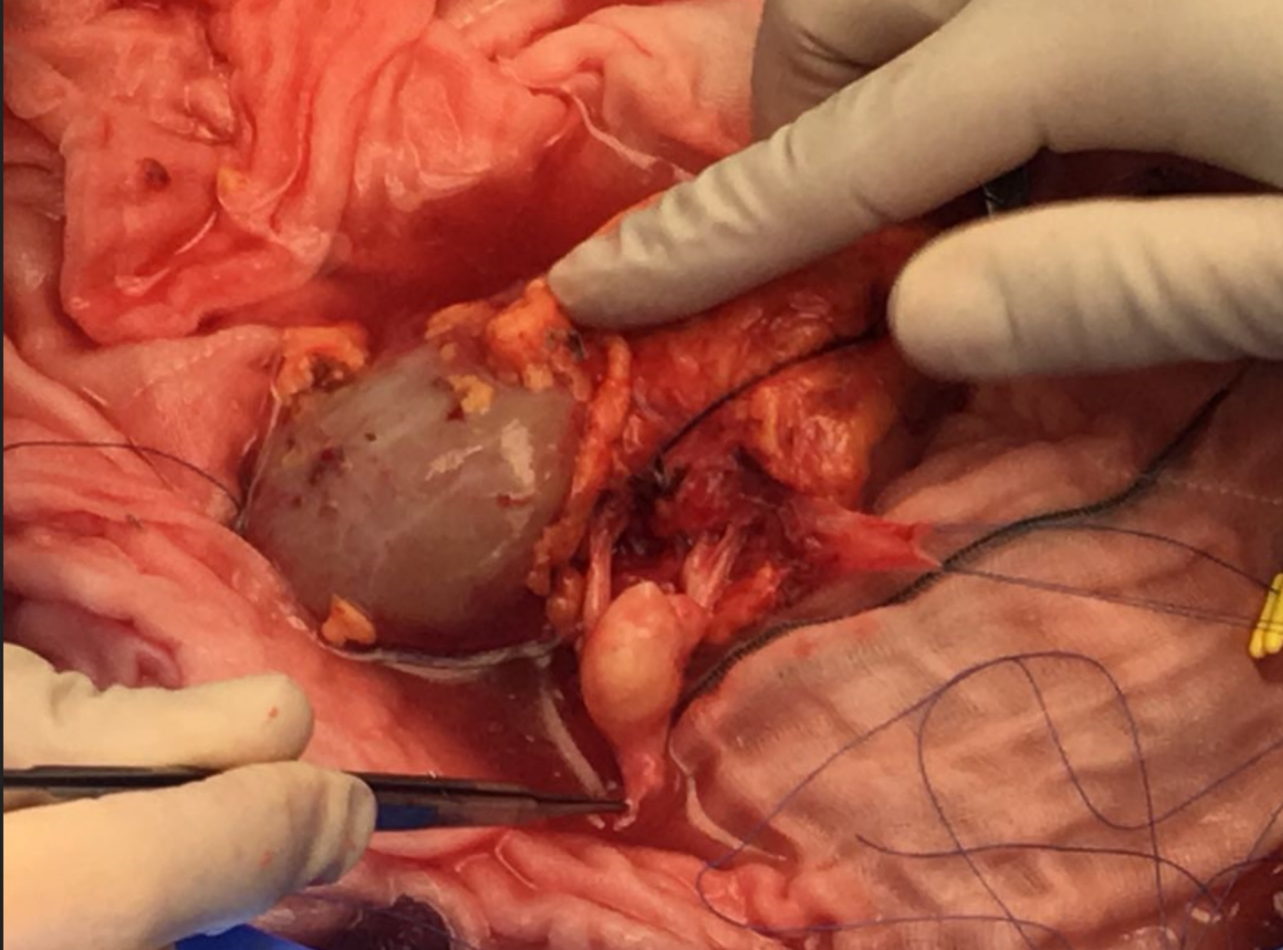
Kidney with the renal artery aneurysm

**Figure 3:**
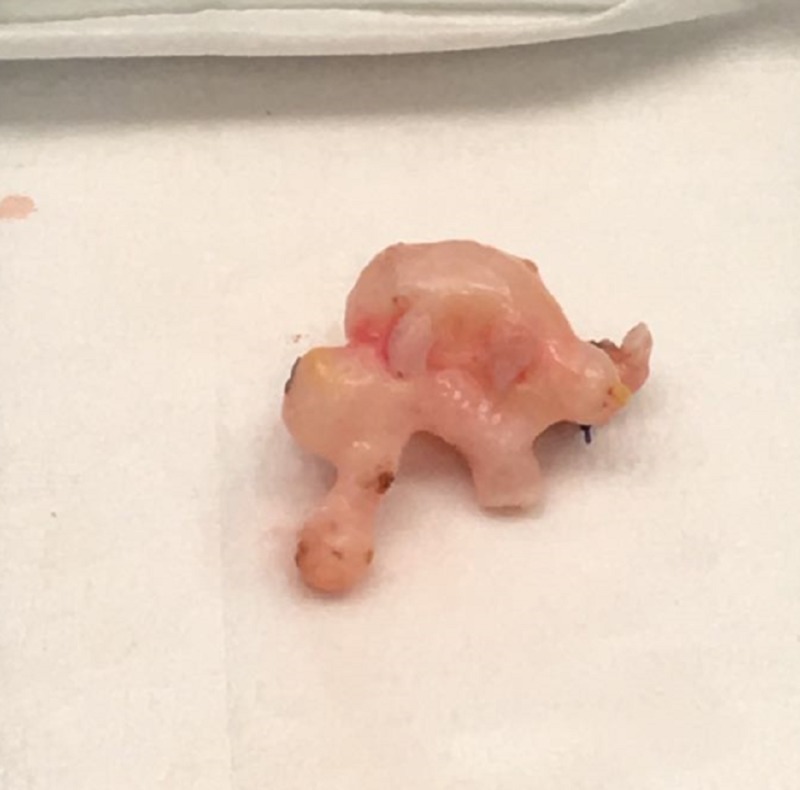
Ex vivo renal artery aneurysm dissected

**Figure 4:**
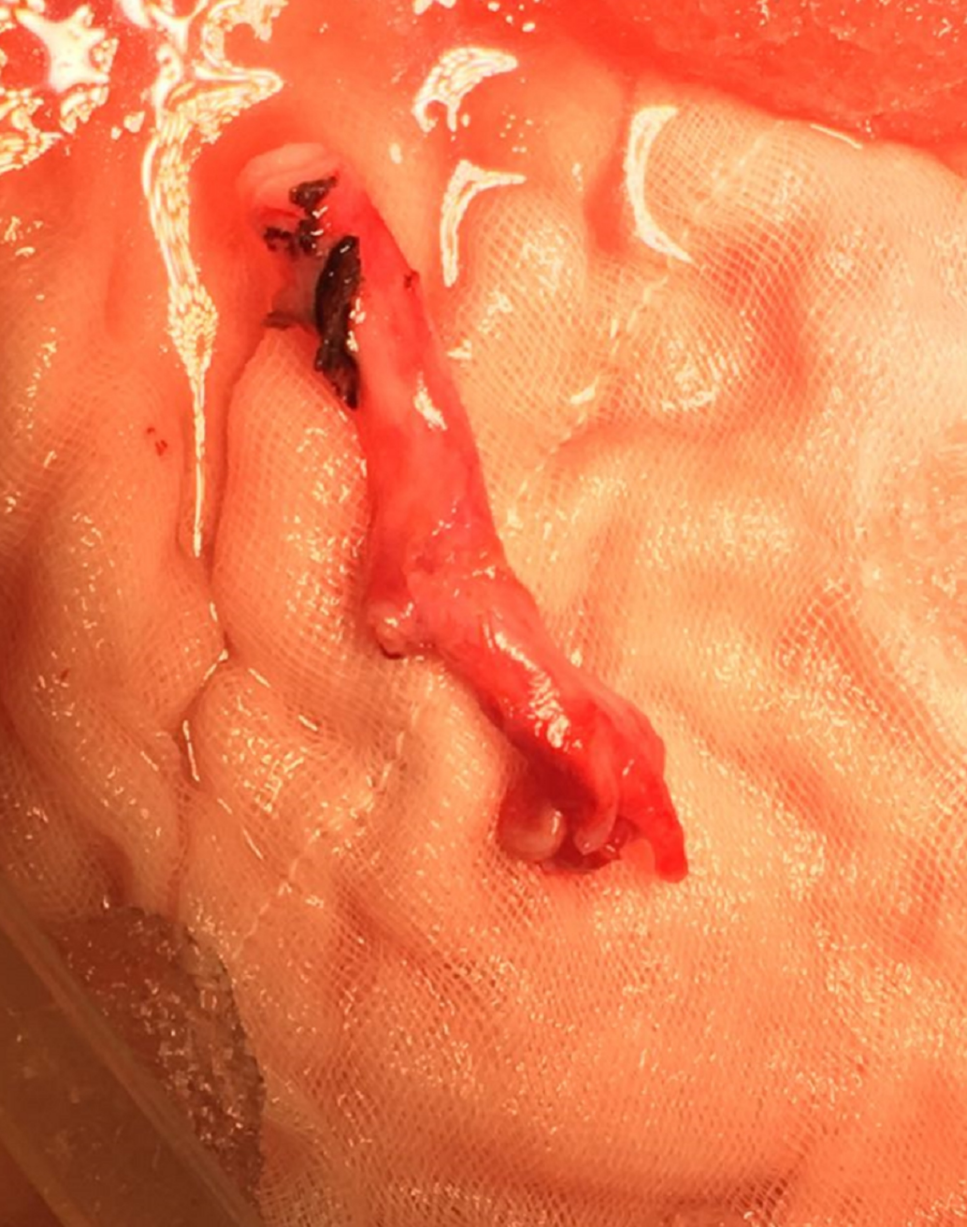
The graft of iliac artery used for renal artery reconstruction

It was decided that an ex vivo laparoscopic reconstruction of the renal artery using a graft from the internal iliac artery was the best approach, as shown in Figures 2-4.

## Discussion

Endovascular surgical techniques may be used in the case of an amenable morphology and with more proximal lesions; however, in cases where the RAAs are more distal and hilar, open surgical procedures are required. In these cases, an ex vivo repair for a complex RAA is a minimally invasive procedure that allows for a smaller incision and, therefore, improved recovery times and reduced incisional morbidity [5].

Successful ex vivo renal artery reconstruction has previously been conducted and reviewed in a number of instances. For example, Ronald et al. reported reconstruction in 24 patients with a history of hypertension. Through this procedure, postoperative tubular necrosis was avoided and when observing the period between six months to six years, 95% of the patients were reported to have been cured or improved [6]. Similarly, Scherrer et al., in continuation of a series of seven successful ex vivo repairs of RAAs in donor kidneys, reported another case of a 66-year-old man, who was hypertensive despite two maintenance medications [5]. The smaller incision through the laparoscopic method allowed for an improved recovery time as well as fewer complications. Even though the patient was hospitalized again with obstipation after two weeks, a duplex ultrasound of the transplanted kidney revealed normal renal function and normal bowel movement returned in 48 hours. Consistent with these cases, our case report also revealed an improved recovery time and a less-invasive reconstruction of the renal artery [7].

## Conclusions

Ex vivo repair and reconstruction of the renal artery provides a great potential for the treatment of most vascular lesions, especially those challenging for endovascular or open surgical options. This can allow not only limiting nephrectomy for only the severely atrophic or infarcted kidneys but also allow for a larger number of kidneys available for transplantation post-repair which would otherwise be discarded.
